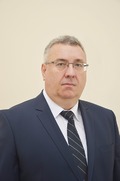# Plant cells and algae in bioreactors III

**DOI:** 10.1002/elsc.201900155

**Published:** 2019-12-04

**Authors:** Atanas Pavlov

**Affiliations:** ^1^ University of Food Technologies The Stephan Angeloff Institute of Microbiology Bulgarian Academy of Sciences Plovdiv Bulgaria

Dear Readers,

It is our pleasure to present the third Special Issue on “Plant cells and algae in bioreactors”. This Special Issue has been compiled based of the success of the previous two, published in 2009 and 2014, and presents recent achievements and the state of‐the‐art in this emerging area of bioprocess engineering.

Over the years after the first Special Issue, the interest in microalgae and plant cells as sources of ingredients for the food, pharmaceutical and cosmetic industries, biofuels and many other domains has been increasing continuously. While, the application of microalgae in various areas like environmental clean‐up and improvement of bioreactor technologies for micropropagation of economically valuable plant species are also currently receiving considerable attention. Thus, the aim of this Special Issue is to present the most recent achievements and research focused on the current status, advancement, and the prospects for further developments in that field.

The advantages of plant in vitro technology for production of bioactive substances are widely discussed. However, the problems with low and unstable yields of target metabolites remain unsolved, despite researchers’ efforts for more than 60 years. This area is addressed in the review presented by Sanchez‐Muñoz et al., who have summarized accumulated knowledge about the relationship between in vitro maintenance and epigenetic changes in plant in vitro systems and the prospects of avoiding decline in metabolite production.

When optimization of the production processes based on plant in vitro systems is discussed, the elicitation approach cannot be omitted. Halder et al. have contributed with an interesting review presenting their point of view about elicitation as a main tool in enhancing the production of secondary metabolites by hairy roots. The authors have paid special attention to the simultaneous application of more than one elicitor as part of the integrated approach for optimization of secondary metabolites production.

Despite the significant progress, bioreactor cultivation and the complex scale‐up remain major limitations for the industrial implementation of plant in vitro systems, as far as the choice of bioreactor type largely depends on the type of the in vitro culture that will be cultivated. Working in this field, Skrzypczak‐Pietraszek and co‐workers present their results on the applicability and prospects of the Platform™ temporary immersion bioreactor and conclude that the Platform™ system capable of simultaneously combining numerous single bioreactors is suitable for scale‐up secondary metabolites production by shoot type plant in vitro cultures. Bioreactor cultivation of shoots is also the subject of the studies presented by other group. Rubio‐Rodríguez et al. have proposed a biotechnological system for secondary metabolites production by *Castilleja tenuiflora* based on temporary immersion cultivation in RITA bioreactors under nitrogen deficiency and addition of exogenous spermine. Finally, the topic on bioreactor design for secondary metabolites production by differentiated plant in vitro systems is closed with the investigation of in situ galanthamine extraction during cultivation of Summer Snowflake shoot culture in a two‐phase bubble column cultivation system by Ivanov and co‐workers, who proposed this cultivation system as an important tool not only to increase yields, but also as an approach for modification of secondary metabolite patterns.

With the development of the so‐called low‐cost bioreactors as well as of the temporary immersion concept, the use of bioreactors in plant biotechnology found a new direction–industrial micropropagation of different plant species of commercial importance. In the Special Issue this area is presented with the valuable review by Vidal and Sánchez, who have summarized the current state‐of‐the‐art and discussed the future prospect of bioreactor technology in the propagation of woody plant species.

The potential of plant in vitro systems for the production of commercially valuable metabolites has been widely discussed over the last two decades. The advantages are clear, but for development of effective processes it is necessary to accumulate a critical mass of data with respect to: 1) different metabolites produced by different plant in vitro systems; 2) the relationship between the degree of differentiation of used plant culture and metabolite yields; 3) development of alternative cultivation techniques and unconventional approaches for process optimization, etc. An important study in this area is presented by Gaid et al. The authors report on the successful transfer of the root cultures of *Hypericum perforatum* from shake flasks to a stirred tank bioreactor and the parameters of the process of hyperforin production, and outlining the possible industrialization in the near future.

A rational approach to optimizing the design of photobioreactors and microalgae cultivation processes is based on the understanding of the physiological situation; gassing, and nutrients optimization. The knowledge about microalgae kinetics and physiology founded on these complex models of photobioreactors and cultivation helps tackle optimization and scale‐up tasks. This aspect is addressed in two valuable reviews presented by Schediwy et al. and Scheufele et al., in which the authors have synthesized the knowledge about microalgae kinetics as a guideline for photobioreactor design and process development, and complex mathematical analysis of a photobioreactor system.

In order to better understand and assess cellular carbon partitioning and carbon economy, it is important to develop suitable methods for quantitating inorganic carbon cycling between photosynthetic microorganisms and their environment. An important contribution in this area is presented by Müller et al. The authors have summarized the complexity and limitations of the detection of inorganic carbon cycling and the recent achievements of the experimental techniques. They have described their point of view about the prospects of both experimental and modeling methods as a basis for correct inorganic carbon cycling.

The focus on the use of microalgae in environmental clean‐up is presented in the review of Wollmann and co‐workers. Given the fact that wastewater clean‐up is a global problem, the authors have summarized extremophilic microalgae‐based wastewater treatment technologies for industrial wastewater sources that have already been put into practice.

For the needs of the optimization of the cultivation processes of plant cells and algae, reliable analytical methods are necessary. In the case of fatty acid production by microalgae, the currently used methods for analysis of triacylglicerides are time‐consuming and laborious. Morschett and co‐workers propose a possible solution, developing a rapid method for analysis of fatty acid produced by microalgae based on the advantages of ToF–MS analytical technique. The authors have demonstrated the applicability of the method through fingerprinting of fatty acids of *Chlorella vulgaris*.

Microalgae biomass contains high amount of proteins and therefore attracted commercial interest. Nowadays used conventional processes for protein extraction are complicated, time consuming, and expensive. Koyande et al. proposed alternative way for protein extraction based on the liquid biphasic flotation. The authors have integrated cell‐disruption method of electrolysis with liquid biphasic flotation systems to extract proteins from *Chlorella vulgaris* and present their results on the optimization of the operating parameters.

The isolation and characterization of novel high‐value compounds from microalgae with a unique highly effective action has been of continuous interest. Nicolova et al. contribute to the Special Issue with their research on the antitumor effect of a heteropolysaccharide biosynthesized by the red microalgae *Porphyridium sordidum*. The authors have demonstrated that the heteropolysaccharide produced in combination with electroporation is effective against a treated model cancer cell line. The interest in the use of algal extracts in agriculture has been growing during the last decade. In the Special Issue this area is presented by Michalak et al. The authors demonstrate the applicability of the combined application of algal extracts and static/alternating magnetic field. This approach is environmentally friendly and eliminates the use of chemical growth regulators.

In the end, I would like to thank all authors for their invaluable contributions and the reviewers for their helpful criticisms. Together we have provided an excellent overview of the current status of plant and algae in vitro technologies. I believe this third Special Issue will be the next step toward the commercial implementation of plant cells and algae in vitro technologies.